# Confidence, Barriers, and Role Identity of General Practice Independent Pharmacist Prescribers in Northern Ireland

**DOI:** 10.3390/healthcare13080933

**Published:** 2025-04-18

**Authors:** Lyndsey Alexander, Kingston Rajiah, Aaron Courtenay, Nermeen Ali, Ahmed Abuelhana

**Affiliations:** 1 Antrim Ballymena GP Federation, Ballymena BT42 1HL, UK; 2 School of Pharmacy and Pharmaceutical Sciences, Ulster University, Coleraine BT52 1SA, UK; a.courtenay@ulster.ac.uk (A.C.); n.ali@ulster.ac.uk (N.A.); a.abuelhana@ulster.ac.uk (A.A.)

**Keywords:** clinical decision-making, independent prescribing, role ambiguity, healthcare workforce integration, mentorship in prescribing

## Abstract

**Background:** The role of General Practice Independent Pharmacist Prescribers (GPIPPs) has expanded significantly in primary care, with increasing responsibilities in medicines optimisation and chronic disease management. However, gaps remain in understanding their confidence in clinical decision-making, the barriers they face, and their professional identity within multidisciplinary teams. This study aimed to explore GPIPPs’ confidence, identify barriers to their prescribing autonomy, and assess the clarity of their role and their support within primary care settings in Northern Ireland. **Methods:** A cross-sectional study design was employed, combining quantitative and qualitative approaches. Data were collected via a Jisc online questionnaire distributed to GPIPPs working in general practices across Northern Ireland. The questionnaire assessed their demographic information, confidence in clinical decision-making, barriers to prescribing, and professional identity. Qualitative data from open-ended responses were analysed using thematic analysis. **Results:** Quantitative findings indicated that most GPIPPs viewed clinical decision-making as integral to their role, yet only a few felt adequately prepared by their independent prescribing courses. Qualitative analysis revealed themes such indemnity concerns, insufficient training, role ambiguity, and variability in GP support. **Conclusions:** The study highlights that while GPIPPs are confident in their prescribing roles, significant barriers such as indemnity concerns, training gaps, and role ambiguity hinder their full potential. Policy-level changes, including clearer indemnity policies, enhanced training programs, and standardised mentorship, are essential to optimise GPIPPs’ integration and effectiveness in primary care. Addressing these challenges will ensure that GPIPPs can fully contribute to patient care as autonomous prescribers.

## 1. Introduction

The role of General Practice Independent Pharmacist Prescribers (GPIPPs) has expanded significantly in primary care, with increasing responsibilities in medicines optimisation, chronic disease management, and clinical decision-making [1]. As healthcare systems shift towards multidisciplinary team (MDT) models, pharmacist prescribers are expected to contribute to medication safety, independent prescribing and patient management [2]. However, despite the growing recognition of pharmacist prescribing, there remains a gap in understanding regarding their confidence in clinical decision-making and the barriers they encounter in this role.

One of the key challenges that general-practice-based pharmacists face is confidence in independent clinical decision-making, particularly when prescribing in primary care settings [3,4]. Research suggests that while pharmacist prescribers demonstrate strong knowledge of pharmacotherapy, they may experience uncertainty when handling complex cases, and regarding legal concerns and professional accountability [5,6]. Factors such as a lack of structured mentorship and variability in general practice (GP) support have been identified as potential contributors to confidence-related barriers [7,8]. Furthermore, role ambiguity within MDTs has been highlighted as a concern, with some pharmacists reporting that their prescribing autonomy is not fully recognised by other healthcare professionals [9].

In addition to confidence barriers, professional indemnity concerns have been reported as a factor for GPIPPs [10]. Pharmacists may hesitate to make high-risk prescribing decisions due to uncertainty about legal protections, leading to a reliance on GP oversight even in cases where independent prescribing is within their scope. Addressing these legal and structural challenges is critical to ensuring that pharmacists can function effectively as independent prescribers.

Moreover, professional identity plays a crucial role in shaping the confidence and autonomy of GPIPPs. Pharmacists who have a well-defined professional identity are more likely to engage confidently in independent prescribing and collaborate effectively within MDTs [9,10]. However, existing prescribing practices, including the level of clinical support and the use of decision-making frameworks, may impact how GPIPPs operate in practice. Policy guidelines related to indemnity, prescribing authority, and interprofessional collaboration establish the structural foundation for independent prescribing, yet variability in their implementation may create uncertainty and limit prescribing confidence. Strengthening professional identity and aligning existing policies and practices more consistently could enhance GPIPP integration and effectiveness within primary care.

While previous research has established the expanding role and growing responsibilities of GPIPPs within primary care, the existing literature remains fragmented regarding the factors affecting their confidence and professional identity across diverse healthcare contexts. There remains considerable uncertainty about the confidence, barriers, and role identity of GPIPPs in Northern Ireland. No studies have directly examined how these contextual variations influence GPIPPs’ experiences, specifically in Northern Ireland. This knowledge gap limits our understanding of the support required for pharmacist prescribers to function optimally in GP settings. The primary research question guiding this study is as follows: What are the key factors influencing GPIPPs’ confidence in clinical decision-making, their perceived barriers, and their professional identity in Northern Ireland’s primary care context? This study hypothesises that GPIPPs in Northern Ireland experience significant variability in confidence and perceived professional identity due to inconsistencies in policy implementation, clinical mentorship availability, and role recognition within multidisciplinary teams.

This research will provide valuable insights into the factors influencing GPIPPs’ confidence and professional identity, highlighting the structural and professional barriers that they face. The findings will inform targeted policy recommendations, training improvements, and support strategies to enhance the role of GPIPPs in primary care settings. The study objective is to explore GPIPPs’ confidence in clinical decision-making, the barriers they encounter, and their professional identity within primary care settings.

## 2. Materials and Methods

### 2.1. Study Design

This study employed a cross-sectional study, integrating both quantitative and qualitative approaches to explore the confidence, barriers, and professional identity of GPIPPs in Northern Ireland. The quantitative component aimed to quantify GPIPPs’ perceptions and experiences, while the qualitative component sought to gather in-depth insights into their views and the challenges they face in primary care settings. As of the study period, there were 317 active GP practices in Northern Ireland; however, specific data on the total number of independent prescribers currently working within GPs across Northern Ireland is not readily available [9]. To ensure broad representation, the distribution of the questionnaire was facilitated through GP networks, extending across the six counties in Northern Ireland.

### 2.2. Sample Size and Sampling Method

Purposeful sampling was performed to select GPIPPs working within GP practices in Northern Ireland. This approach ensured that only those GPIPPs were included, allowing for the collection of meaningful and representative data. Pharmacists working in other sectors, such as hospital or community pharmacies, were excluded to maintain the study’s focus on the primary care setting. Although no formal power calculation was conducted, the sample size was sufficient to identify key trends and themes related to GPIPPs’ confidence, barriers, and professional identity in clinical decision-making. The questionnaire was sent to 50 GPIPPs, and we received 37 responses, resulting in a response rate of 74%. Of the 37 responses, all were deemed complete and suitable for analysis.

### 2.3. Questionnaire

The questionnaire was developed based on the existing literature [11,12,13]. It consisted of four sections with a total of 27 questions (refer to the Supplementary Materials): Section 1 covered demographics and professional background (5 items); Section 2 assessed confidence in clinical decision-making (7 items); Section 3 explored barriers to confidence (4 items); and Section 4 examined professional identity and clinical support (11 items, including open-ended qualitative responses). Face validity was carried out through a subjective evaluation to determine whether the questionnaire appeared to measure that which was intended. Experts (Lead Practice Pharmacists) from the Antrim/Ballymena Federation in Northern Ireland reviewed the questionnaire to assess whether the items related to the construct being measured and whether the questions were worded clearly and appropriately. Experts provided feedback on whether any questions were irrelevant, unclear, or misleading and suggested revisions as needed. Based on the feedback from experts, the questionnaire was revised to improve clarity and alignment.

### 2.4. Data Collection

Data were collected anonymously using the Joint Information Systems Committee (Jisc)^®^ online survey platform (https://www.ulster.ac.uk/ds/staff/jisc-online-surveys, accessed on 1 April 2025). Participation was voluntary, and informed consent was obtained electronically before respondents could proceed with the survey. Participants were assured of confidentiality, and data were stored securely, accessible only to the research team. The study adhered to the Declaration of Helsinki and the guidelines set by the ethical review board, ensuring that responses were collected and used exclusively for research purposes, with participant confidentiality and anonymity maintained throughout.

### 2.5. Data Analysis

Data analysis was conducted using both quantitative and qualitative approaches. Quantitative data were analysed using Microsoft Excel, where descriptive statistics (frequencies, percentages, and measures of central tendency) were calculated to summarise participant responses. Qualitative data from open-ended responses were analysed using Braun and Clarke’s thematic analysis framework. The analysis followed a structured six-step approach [14]. First, all qualitative data were carefully read multiple times to gain a clear understanding. Next, the data were organised through coding, condensing large volumes of information into smaller, meaningful segments. In the third step, codes were examined and grouped into preliminary themes specific to the study. These themes were then reviewed, refined, and further developed to ensure coherence and relevance. In the fifth step, themes and subthemes were carefully defined, clarifying their relationships and interactions. Finally, the integrity of the data was maintained by clustering themes and synthesising findings, with an experienced qualitative researcher independently reviewing the themes to ensure rigour and validity.

## 3. Results

All quantitative findings presented are descriptive, with percentages rounded to whole numbers due to the small sample size. Given the limited sample size (n = 37), formal statistical testing was not conducted, and these results should be interpreted as being illustrative rather than inferential.

### 3.1. Quantitative Results

#### 3.1.1. Demographics and Professional Background

A total of 37 GPIPPs responded to this study. Table 1 summarises the demographics and professional background of respondents. The majority of respondents were female. Most of the pharmacists had been working as pharmacists for over 10 years. Regarding experience as a GPIPP, some had worked in this role for 4–6 years, while some had been in the role for more than 10 years. More than half of the respondents reported working part-time.

#### 3.1.2. Confidence in Clinical Decision-Making

Table 2 summarises the GPIPPs’ confidence in clinical decision-making. Most of the respondents strongly agreed or agreed that clinical decision-making is an integral part of their role as independent prescribers. However, despite this recognition, only a few felt that their independent prescribing course had adequately prepared them for clinical decision-making, with a substantial proportion agreeing with but not strongly supporting this statement. Notably, confidence in making clinical decisions within their boundaries of competence was high, with most of the respondents strongly agreeing or agreeing. Similarly, most of the pharmacists reported comfort in making therapeutic decisions. While the majority felt confident in applying their prescribing knowledge, one-third believed that the responsibility for making a clinical decision lies solely with the prescriber who signs the prescription, whereas some either disagreed or were neutral. This suggests an awareness of the shared and multidisciplinary nature of prescribing decisions in general practice.

#### 3.1.3. Barriers to Confidence in Clinical Decision-Making

Table 3 summarises the GPIPPs’ barriers to confidence in clinical decision-making. Several factors were identified as barriers to pharmacists’ confidence in clinical decision-making. Nearly half of the respondents cited indemnity concerns as a major factor limiting their confidence. Insufficient training was another key issue, reported by some of the pharmacists, while a few highlighted personal confidence issues as a limitation. Additionally, very few respondents felt that inadequate clinical support affected their confidence, and very few reported time constraints as a contributing factor. These findings suggest that external factors, such as legal and institutional support, play a significant role in shaping pharmacists’ confidence, alongside internal factors like training and self-assurance.

#### 3.1.4. Support and Role Clarity

Table 4 summarises the GPIPPs’ satisfaction with clinical support and role clarity. More than three-quarters of pharmacists were either satisfied or very satisfied with the clinical support available in their practice. However, very few respondents expressed dissatisfaction, indicating that while most pharmacists feel supported, some may require additional mentorship or structured decision-making frameworks. When assessing professional role clarity, most of the respondents strongly agreed or agreed that they had a clear understanding of their role as independent prescribers within their workplace. However, a few were either neutral or disagreed, suggesting that role ambiguity still exists for some pharmacists. Additionally, most of the respondents felt that their job description clearly defined the scope of their prescribing activities, while a small proportion did not find this to be the case. These findings indicate that while the majority of GPIPPs are confident in their role, there is a need for further clarity and awareness in certain practice settings (Table 5).

### 3.2. Qualitative

#### 3.2.1. Indemnity Concerns

A key concern among pharmacists was the impact of legal restrictions and liability on their confidence in prescribing. Many pharmacists expressed hesitation in making complex prescribing decisions due to uncertainty about their indemnity coverage, which led to a risk-averse approach.

Supporting quote: “*Indemnity limitations restrict confidence, especially in complex cases*”. This suggests that uncertainty around professional liability may be limiting the scope of independent prescribing.

Closely related to this was hesitancy in prescribing outside of the guidelines, as pharmacists feared the potential consequences of deviating from established protocols. Some participants felt that their prescribing autonomy was constrained by a lack of clarity regarding legal protections.

Supporting quote: “*I avoid prescribing outside guidelines due to uncertainty about legal implications*”.

This highlights the need for clearer indemnity policies and legal guidance to support independent prescribing decisions.

#### 3.2.2. Training and Education Gaps

Many pharmacists highlighted inadequate preparation in independent prescribing courses, stating that their training was largely theoretical and lacked exposure to real-world clinical decision-making.

Supporting quote: “*Training should be more tailored to real-world decision-making rather than just guideline adherence*”.

This indicates a gap between formal training and the complex, nuanced decisions required in practice.

Additionally, there was a clear need for more real-world decision-making training, as many respondents felt underprepared for scenarios that fell outside of strict prescribing guidelines.

Supporting quote: “*I often feel underprepared when dealing with cases that fall outside standard protocols*”.

These findings suggest that incorporating case-based learning, simulation exercises, and hands-on mentorship could better prepare pharmacists for independent prescribing roles.

#### 3.2.3. Role Ambiguity and Recognition

The misunderstanding of GPIPPs’ roles within healthcare teams was a recurring theme, with some pharmacists feeling that their contributions were not fully recognised. Many reported that colleagues, particularly GPs, did not fully understand the extent of their prescribing authority, which impacted their confidence and ability to work autonomously.

Supporting quote: “*We are expected to be ‘script checkers’ rather than true clinical decision-makers*”.

This was further reinforced by perceptions of being seen as ‘script checkers’ rather than decision-makers. Several pharmacists expressed frustration that despite their independent prescriber status, their role was often viewed as supportive rather than autonomous.

Supporting quote: “*GPs often misunderstand our prescribing autonomy, which affects confidence*”.

These findings highlight the need for greater interprofessional education and role advocacy to improve the recognition and integration of GPIPPs in primary care teams.

#### 3.2.4. GP Support Variability

Pharmacists reported variability in GP support, with some benefiting from strong mentorship from GPs, which enhanced their confidence and prescribing ability.

Supporting quote: “*Some GPs provide excellent clinical mentorship, which improves confidence*”.

This suggests that having structured support networks within GPs plays a critical role in reinforcing pharmacists’ autonomy.

However, a lack of structured support in complex cases was a significant concern for others. Some pharmacists felt isolated when making complex prescribing decisions without clear guidance, leading to uncertainty and reduced confidence.

Supporting quote: “*Others expect us to manage complex cases with little guidance*”.

These findings suggest that standardised mentorship programs and clearer collaborative frameworks could help ensure that all GPIPPs receive the support they need.

## 4. Discussion

This study explored the confidence of GPIPPs in clinical decision-making, the barriers they face, and their professional identity within primary care settings. The findings revealed that most GPIPPs felt confident in making clinical decisions within their competencies. Several GPIPPs reported that their independent prescribing course adequately prepared them for real-world decision-making. However, a gap between theoretical training and practical experience emerged, with several GPIPPs noting a need for more case-based, real-world training. This gap suggests that while initial training may provide the foundational knowledge, it does not fully prepare prescribers for the complexities of clinical practice. The findings highlight that simulation-based learning and practical mentorship could be key to enhancing GPIPPs’ confidence in handling complex cases and making autonomous clinical decisions [15,16].

Several barriers to confidence in prescribing were identified, with insufficient indemnity coverage being a prominent concern. Some GPIPPs expressed fears about legal liability when making complex prescribing decisions. This echoes findings in the existing literature where the perception of risk and the fear of professional repercussions limit prescribing autonomy [17]. This issue suggests a need for clearer legal protections and comprehensive indemnity coverage tailored to independent prescribers. Additionally, a lack of exposure to complex patient cases was another barrier, with some GPIPPs indicating uncertainty when prescribing outside of established guidelines. This hesitancy highlights the importance of providing decision-making frameworks and more exposure to challenging clinical situations during training to improve pharmacist prescribers’ confidence and competence in diverse scenarios [18,19].

The study found that while many GPIPPs had a clear professional identity, some experienced role ambiguity within primary care teams. Many GPIPPs reported being viewed primarily as ‘script checkers’ rather than clinical decision-makers. This aligns with previous research indicating that independent pharmacist prescribers often face difficulties in being recognised as autonomous prescribers due to entrenched perceptions within MDTs [13,14]. The lack of clarity regarding their role hinders full integration into MDTs and reduces their ability to exercise their prescribing autonomy [20]. Addressing this challenge requires improved interprofessional education, better-defined roles, and targeted awareness campaigns to promote the role of pharmacist prescribers as clinical decision-makers.

This study highlighted variability in the level of support provided by GPs, which had a significant impact on GPIPPs’ confidence and effectiveness in prescribing. While some GPIPPs reported benefiting from strong mentorship, others indicated that they were expected to manage complex cases with little guidance or support. This inconsistency in supervision and mentorship highlights the need for standardised mentorship programs within primary care settings. Those who received structured mentorship and opportunities for collaborative practice with GPs were more confident in their prescribing roles [21,22]. Conversely, those without adequate support expressed hesitation in managing more challenging clinical situations. These findings highlight the importance of collaborative decision-making models to ensure that GPIPPs have the guidance they need to perform effectively in their roles [23,24].

### 4.1. Implications

This study’s findings highlight the urgent need for policy-level changes to support GPIPPs in overcoming structural barriers such as indemnity concerns, a lack of standardised mentorship, and unclear role definitions. Several policy-driven solutions could enhance pharmacist prescribers’ confidence, including establishing clearer indemnity policies to ensure pharmacists feel legally protected when making independent prescribing decisions; incorporating case-based learning, simulation exercises, and interprofessional education into independent prescribing courses; developing formal mentorship programs within general practice settings to ensure pharmacists receive consistent supervision and guidance; and conducting awareness campaigns to educate healthcare professionals and the public on the scope and value of independent pharmacist prescribers in primary care settings.

### 4.2. Strengths and Limitations

This study provided rich, contextually grounded insights into GPIPPs’ confidence, professional identity, and perceived barriers in clinical decision-making. The qualitative component was strengthened by achieving thematic saturation, enhancing the validity and credibility of the findings. Purposeful sampling ensured that participants had relevant experience as independent prescribers, enabling meaningful data collection that was aligned with the study’s objectives. The relatively small sample size (n = 37) limits the quantitative representativeness and generalisability of these findings to the broader population of GPIPPs in Northern Ireland. Due to the purposeful sampling approach and absence of formal statistical testing, percentages have been presented descriptively without decimals to avoid implying undue precision. Hence, the quantitative results should be interpreted as illustrative patterns rather than definitive statistical evidence. Nevertheless, the qualitative component achieved thematic saturation, significantly strengthening the validity and credibility of the findings. Therefore, the study should be understood primarily as a qualitative investigation, enriched by descriptive quantitative data that provide additional context and support.

### 4.3. Future Research Directions

Future research should explore the long-term impact of structured mentorship programs and simulation-based learning on GPIPPs’ confidence and prescribing practices. Investigating the effectiveness of various indemnity coverage models, specifically tailored for independent prescribers, could also provide valuable insights into how legal protections influence prescribing autonomy. Further studies should examine the perceptions of other healthcare professionals, such as GPs and nurses, regarding the role of pharmacist prescribers, to better understand the dynamics of multidisciplinary teams and identify strategies to improve role integration. Additionally, research into the barriers and facilitators of interprofessional education for GPIPPs could offer practical solutions for enhancing collaborative decision-making in primary care. Finally, studies exploring the influence of case-based training and exposure to complex clinical situations on decision-making confidence could help inform curriculum design and policy recommendations for independent prescribing courses.

## 5. Conclusions

The quantitative and qualitative findings revealed that while GPIPPs demonstrate high confidence in prescribing within defined competencies, significant barriers persist in handling complex cases, overcoming legal concerns, and gaining professional recognition. Addressing these issues through policy changes, structured training, and better collaboration within MDTs will be essential in optimising the role of GPIPPs and ensuring their full integration into primary care prescribing pathways.

## Figures and Tables

**Table 1:** Demographics and Professional Background of GPIPPs.

Characteristic	n	Percentage (%)
Gender		
Female	28	76
Male	9	24
Age group (Years)		
21–30	2	5
31–40	16	43
41–50	14	38
51–60	5	14
Over 60	0	0
Years worked as a pharmacist		
1–3	0	0
4–6	2	5
7–9	4	11
More than 10	31	84
Years Worked as a GPIPP		
1–3 years	10	27
4–6 years	13	35
7–9 years	2	5
More than 10 years	12	33
Employment Type		
Full-time	16	43
Part-time	21	57

**Table 2:** Confidence in Clinical Decision-Making.

Statement	Strongly Agree (n, %)	Agree (n, %)	Neither Agree nor Disagree (n, %)	Disagree (n, %)	Strongly Disagree (n, %)
Clinical decision-making is part of the role	35 (95%)	2 (5%)	0 (0%)	0 (0%)	0 (0%)
The independent prescribing course fully prepared me	17 (47%)	15 (40%)	1 (3%)	2 (5%)	2 (5%)
I am confident in decisions within my competence	36 (97%)	1 (3%)	0 (0%)	0 (0%)	0 (0%)
I am comfortable making therapeutic decisions	34 (92%)	2 (5%)	1 (3%)	0 (0%)	0 (0%)
I am aware of my prescribing limitations	27 (73%)	10 (27%)	0 (0%)	0 (0%)	0 (0%)
Clinical decision-making is multidisciplinary	27 (73%)	10 (27%)	0 (0%)	0 (0%)	0 (0%)
Responsibility lies wholly with prescriber	17 (47%)	15 (40%)	1 (3%)	2 (5%)	2 (5%)

**Table 3:** Barriers to Confidence in Clinical Decision-Making.

Barrier	n	Percentage (%)
Insufficient indemnity coverage	18	49%
Lack of training	11	31%
Confidence issues	8	22%
Limited clinical support	4	11%
Time constraints	2	5%

**Table 4:** Satisfaction with Clinical Support and Role Clarity.

Statement	Strongly Agree (n, %)	Agree (n, %)	Neither Agree nor Disagree (n, %)	Disagree (n, %)	Strongly Disagree (n, %)
Satisfied with clinical support	10 (27%)	19 (51%)	4 (11%)	4 (11%)	0 (0%)
Role identity is clear in my workplace	11 (30%)	18 (49%)	3 (8%)	3 (8%)	2 (5%)
My job description defines my prescribing scope	12 (33%)	18 (49%)	3 (8%)	2 (5%)	2 (5%)

**Table 5:** Themes, Sub-Themes, and Supporting Quotes from Qualitative Analysis.

Themes	Sub-Themes	Supporting Quotes
Indemnity Concerns	Legal restrictions and fear of liability	“Indemnity limitations restrict confidence, especially in complex cases”.
	Hesitancy in prescribing outside guidelines	“I avoid prescribing outside guidelines due to uncertainty about legal implications”.
Training and Education	Inadequate preparation in independent prescribing courses	“Training should be more tailored to real-world decision-making rather than just guideline adherence”.
	Need for more real-world decision-making training	“I often feel underprepared when dealing with cases that fall outside standard protocols”.
Role Ambiguity	Misunderstanding of GPIPP roles within healthcare teams	“We are expected to be ‘script checkers’ rather than true clinical decision-makers”.
	Perceived as ‘script checkers’ rather than decision-makers	“GPs often misunderstand our prescribing autonomy, which affects confidence”.
GP Support Variability	Strong mentorship from some GPs	“Some GPs provide excellent clinical mentorship, which improves confidence”.
	Lack of structured support in complex cases	“Others expect us to manage complex cases with little guidance”.

## Data Availability

Data presented in the study are stored securely at school of Pharmacy and Pharmaceutical Sciences, Ulster University. The investigators act as custodians for the data processed and generated by this study and they are also responsible for the access to any information included. Data are available upon request from the corresponding author. Due to privacy and institutional regulations, the data are not publicly accessible.

## Supplementary Materials

The following supporting information can be downloaded at: https://www.mdpi.com/article/10.3390/healthcare13080933/s1, Questionnaire.
