# Supplementary material for: Confidence, Barriers, and Role Identity of General Practice Independent Pharmacist Prescribers in Northern Ireland
Source: Healthcare (Basel). 2025 Apr 18;13(8):933. doi: 10.3390/healthcare13080933 (PMC12027101; doi:10.3390/healthcare13080933)
Supplement: Supplementary file 1 [file healthcare-13-00933-s001.zip › healthcare-3524118-supplementary.pdf]

1. What is your gender?
    - Male
    - Female
    - Other
    - Prefer not to say
2. What is your age group?
    - 21 – 30 years
    - 31 – 40 years
    - 41 – 50 years
    - 51 – 60 years
    - Over 60 years
3. How many years have you been qualified as a pharmacist?
    - 1 – 3 years
    - 4 – 6 years
    - 7 – 9 years
    - More than 10 years
4. How many years have you worked as a General Practice Independent Pharmacist Prescriber (GPIPP)?
    - 1 – 3 years
    - 4 – 6 years
    - 7 – 9 years
    - More than 10 years
5. What is your current employment type?
    - Full-time (37.5 hours per week)
    - Part-time (less than 37.5 hours per week)

Please indicate your level of agreement with the following statements:

6. Clinical decision-making is part of the independent pharmacist prescriber role.
    - Strongly agree
    - Agree
    - Neither agree nor disagree
    - Disagree
    - Strongly disagree
7. The independent prescribing course fully prepared me for the clinical decision-making process.
    - Strongly agree
    - Agree
    - Neither agree nor disagree
    - Disagree
    - Strongly disagree
8. I am confident in making clinical decisions within my boundaries of competence.
    - Strongly agree
    - Agree
    - Neither agree nor disagree
    - Disagree
    - Strongly disagree
9. I am comfortable making therapeutic decisions about patient treatment.
    - Strongly agree
    - Agree
    - Neither agree nor disagree
    - Disagree
    - Strongly disagree

10. I am aware of my limitations as a prescriber.
    - Strongly agree
    - Agree
    - Neither agree nor disagree
    - Disagree
    - Strongly disagree
11. Clinical decision-making is a multidisciplinary process.
    - Strongly agree
    - Agree
    - Neither agree nor disagree
    - Disagree
    - Strongly disagree
12. The responsibility for the clinical decision lies wholly with the person who signs the prescription.
    - Strongly agree
    - Agree
    - Neither agree nor disagree
    - Disagree
    - Strongly disagree

13. What factors contribute to a lack of confidence in clinical decision-making? (Select all that apply)

- Lack of training
- Insufficient professional indemnity coverage
- Limited clinical support
- Confidence issues
- Time constraints

- Other (please specify)

14. I require further training to develop confidence in clinical decision-making.
    - Strongly agree
    - Agree
    - Neither agree nor disagree
    - Disagree
    - Strongly disagree
15. I require further experience to develop confidence in clinical decision-making.
    - Strongly agree
    - Agree
    - Neither agree nor disagree
    - Disagree
    - Strongly disagree
16. My job description clearly defines the scope of my prescribing activities.
    - Strongly agree
    - Agree
    - Neither agree nor disagree
    - Disagree
    - Strongly disagree
17. I have a clear identity of my role as an independent pharmacist prescriber in my workplace.
    - Strongly agree
    - Agree
    - Neither agree nor disagree
    - Disagree

- Strongly disagree

18. Are you satisfied with the clinical support you have in practice to make clinical decisions?
    - Very satisfied
    - Satisfied
    - Neither satisfied nor dissatisfied
    - Dissatisfied
    - Very dissatisfied
19. If not satisfied, please provide details about the barriers to clinical support. *(Open-ended response)*
20. Compared to other prescribers, do you think pharmacists are more or less confident in prescribing in situations where there is uncertainty or complexity (e.g., prescribing outside guidelines or protocols)?
    - More confident
    - Less confident
    - Same confidence level
    - Please explain: *(Open-ended response)*
21. Do you often seek advice from a GP when making clinical decisions?
    - Yes
    - No
    - If yes, in what situations do you seek GP advice? *(Open-ended response)*
22. In the clinical decision-making process, does the GP have an overseeing role in the multidisciplinary team?
    - Yes
    - No
    - Please explain: *(Open-ended response)*
23. Do you think the independent pharmacist prescriber has a role in diagnosis?

- Yes
- No
- If not, what barriers prevent pharmacists from undertaking this role? *(Open-ended response)*

24. Do you think the public is aware of the role of independent pharmacist prescribers?
    - Yes
    - No
    - Please explain: *(Open-ended response)*
25. Do you have any comments on the General Practice Pharmacist's professional identity within the workplace? *(Open-ended response)*
26. In regard to the clinical decision-making process, how does the professional identity of the General Practice Pharmacist impact decision-making? *(Open-ended response)*
27. Do you have any other comments on the role of independent pharmacist prescribers? *(Open-ended response)*
